# Fucoidan Oligosaccharide Supplementation Relieved Kidney Injury and Modulated Intestinal Homeostasis in D-Galactose-Exposed Rats

**DOI:** 10.3390/nu17020325

**Published:** 2025-01-17

**Authors:** Jing Shi, Yan Xu, Kening Zhang, Ying Liu, Nan Zhang, Yabin Zhang, Huaqi Zhang, Xi Liang, Meilan Xue

**Affiliations:** 1 Institute of Nutrition and Health, School of Public Health, Qingdao University, 308 Ningxia Road, Qingdao 266021, China; 2022028378@qdu.edu.cn; 2 Department of Nutrition and Food Hygiene, School of Public Health, Qingdao University, 308 Ningxia Road, Qingdao 266071, China; 2021010149@qdu.edu.cn (Y.X.); 2024010107@qdu.edu.cn (K.Z.); zhangnan64@qdu.edu.cn (N.Z.); 2021021112@qdu.edu.cn (Y.Z.); huaqi_erin_qy@qdu.edu.cn (H.Z.); liangxi@qdu.edu.cn (X.L.); 3 Laboratory of Cell and Molecular Biology, School of Basic Medicine, Qingdao University, 308 Ningxia Road, Qingdao 266071, China; liuying1@qdu.edu.cn; 4 Department of Biochemistry and Molecular Biology, School of Basic Medicine, Qingdao University, 308 Ningxia Road, Qingdao 266071, China

**Keywords:** fucoidan oligosaccharide, kidney injury, mitochondrial autophagy

## Abstract

**Background/Objectives:** A fucoidan oligosaccharide (FOS), a potent compound derived from algae, is known for its diverse biological activities, including prebiotic activity, anticancer activity, and antioxidative properties, and has demonstrated supportive therapeutic effects in treating kidney ailments. This study was conducted to explore the protective influence of FOS on kidney damage due to aging induced by D-galactose in Sprague Dawley (SD) rats. **Methods:** The low-dose FOS group was administered FOS (100 mg/kg) by gavage, and the high-FOS group received FOS (200 mg/kg) by gavage. **Results:** The findings showed that FOS could effectively mitigate kidney damage and improve the pathological condition of kidney tissues caused by D-gal and enhance kidney function. Intervention with FOS significantly reduced serum creatinine, serum uric acid, and serum urea nitrogen levels, compared to the model group. The protective mechanism of FOS on D-gal-induced kidney injury may be to inhibit oxidative stress and improve impaired mitochondrial function by downregulating the AMPK/ULK1 signaling pathway. FOS could also modulate the expression of mitochondrial autophagy-related proteins (Beclin-1, P62, and LC3II/LC3I), thereby mitigate D-gal-induced excessive mitophagy in the kidney. Furthermore, FOS may protect against kidney injury by preserving intestinal homeostasis. FOS decreased serum lipopolysaccharide levels and enhanced intestinal mucosal barrier function. FOS upregulated the abundances of *Bacteroidota*, *Muribaculaceae*, and *Lactobacillus*, while it decreased the abundances of *Firmicutes*, *NK4A136_group*, and *Lachnospiraceae_NK4A136_group*. FOS supplementation modulated gut microbiota composition, increasing beneficial bacteria and reducing detrimental ones, potentially contributing to improved kidney function. **Conclusions:** FOS may safeguard against renal injury in D-gal-exposed rats by inhibiting kidney excessive mitophagy, preserving mitochondrial function, and regulating intestinal homeostasis.

## 1. Introduction

Aging is a natural process that leads to systemic changes, resulting in reduced organ function and a higher likelihood of age-related diseases, ultimately leading to death [1]. During the aging process, the kidney experiences a gradual decline in function along with histological changes. Aging heightens vulnerability to renal injury, leading to incomplete recovery and progressive kidney dysfunction [2,3,4]. Research indicates that oxidative stress, mitochondrial impairment, and autophagic dysfunction are prevalent mechanisms in age-related kidney damage. Kidney damage may occur due to lipid peroxidation, DNA damage, and protein modification, resulting from high reactive oxygen species (ROS) concentrations or inadequate antioxidant defense [5]. Excessive ROS can damage mitochondria, leading to reduced mitochondrial membrane potential (MMP) and resulting in mitochondrial dysfunction [6]. The kidney’s significant energy requirements are mainly fulfilled by generating adenosine 5′-triphosphate (ATP). Mitochondrial dysfunction impairs ATP production and plays a crucial role in the development of various kidney diseases [7,8]. Mitophagy, a selective autophagic process targeting damaged or surplus mitochondria, is crucial for maintaining cellular energy balance and functionality [9,10]. However, excessive mitophagy can lead to normal mitochondria being encapsulated by autophagosomes for degradation, which affects the normal function of mitochondria and is associated with cell death [11,12]. Recent studies increasingly demonstrate the intrinsic connections between alterations in intestinal flora and kidney pathophysiology. Examples include assessing the direct role of the microbiome in kidney function in germ-free or antibiotic-treated animals and studying the effects of specific microbiomes on kidney health through fecal transplantation [13,14,15]. Therefore, effectively regulating oxidative stress, improving excessive mitophagy, and regulating gut microbial homeostasis may be potential ways to treat age-related kidney injury. 

Prolonged D-gal administration replicates features of natural aging. D-gal, a natural reducing sugar, can be transformed into aldose and hydroperoxide in the presence of high oxidase concentrations, generating superoxide anions and oxygen free radicals. This leads to excessive ROS accumulation in the kidneys, reducing antioxidant enzyme levels and triggering inflammatory responses [16,17]. The oxidative stress caused by D-gal can lead to cellular apoptosis, necrosis, kidney dysfunction, and other types of injuries [18]. Although D-galactose-induced aging mice are widely used because of its simplicity and good performance, this model has limitations in fully recapitulating the complexity of age-related kidney disease, such as its focus on oxidative stress and potential lack of other age-related pathologies.

Fucoidan, a sulfated polysaccharide present in brown algae and echinoderms, demonstrates various pharmacological effects such as antioxidant, anti-inflammatory, and immunomodulatory properties [19,20,21]. Research on this marine-derived natural compound in animals and humans indicates its accumulation in the kidneys, with detectable levels in serum and urine [21,22,23]. Research has found that fucoidan can alleviate oxidative damage in kidney tissues through its antioxidant, anti-inflammatory, and anti-apoptotic activities [24,25,26]. In vitro experiments also revealed that fucoidan could target autophagy signaling pathways to improve aging in human proximal kidney tubular epithelial cells [27]. Our previous research demonstrated that fucoidan alleviates renal injury by modulating the gut–kidney axis in hyperuricemia mice [28]. Fucoidan’s use is restricted by its molecular weight. Fucoidan oligosaccharides (FOSs) exhibit protective effects in mouse models of acute liver injury induced by carbon tetrachloride and D-GalN, as evidenced by a significant reduction in serum malondialdehyde levels and a notable increase in superoxide dismutase and glutathione peroxidase activities [29,30]. The potential of FOS to mitigate age-related kidney damage by reducing oxidative stress, regulating excessive mitophagy, and influencing gut microbiota in D-gal-exposed rats remains unreported.

This study aimed to explore the protective effects and underlying mechanisms of FOS on kidney injury in D-gal-exposed rats, offering a potential intervention target for future research on age-related kidney damage.

## 2. Materials and Methods

### 2.1. Drugs and Reagents

FOSs (90% purity, molecular weight < 10 kDa) were extracted and purified from Chorda filum and purchased from Shanghai Jinpan Biotechnology Co., Ltd. (Shanghai, China). The total sugar content of the fucooligosaccharides was 42.3% by the phenol-sulfuric acid method, and the sulfate group content was 22.5% by the barium chloride gelatin method. The monosaccharide composition of fucooligosaccharides, fucose at 74.8%, mannose, and D-gal (≥99% purity), was acquired from Sigma-Aldrich Chemical Co. (St. Louis, MO, USA). All chemicals and reagents used were of an analytical grade, guaranteeing consistency and reliability in the experiments.

### 2.2. Animal and Experimental Treatment

Forty male Sprague Dawley rats, aged 8 weeks and weighing between 200 and 250 g, were acquired from Beijing Vital River Laboratory Animal Technology Co., Ltd. (Beijing, China) (SCXK [Jing] 2021-0006). Rats were maintained under SPF conditions with unrestricted access to food and water, a 12 h light–dark cycle, temperatures of 22–25 °C, and 50–60% relative humidity. Animal experiments were approved by the Animal Care and Use Committee of the Medical College at Qingdao University, following the National Institutes of Health guidelines for laboratory animal handling and use (approval number: No. 20211008SD4020211215130; approval date: 23 September 2021).

Following a week of adaptive feeding, the animals were randomly assigned to four groups (*n* = 10 each): the control group (CON), model group (MOD), low-dose FOS group (LF), and high-dose FOS group (HF). Rats in the MOD, LF, and HF groups received a subcutaneous injection of D-gal (150 mg/kg) into the neck and back. Concurrently, rats in the MOD group received normal saline by gavage, the LF group was administered FOS (100 mg/kg) by gavage, and the HF group received FOS (200 mg/kg) by gavage. Rats in the CON group received equivalent doses of normal saline via gavage and subcutaneous injection. The 8-week intervention allowed rats unrestricted access to food and water. The rats’ body weights were recorded weekly at a consistent time during the experiment.

After the last intervention, the blood samples, kidney tissue, ileum tissue, and intestinal contents of rats were collected after being anesthetized. The kidney index was determined by dividing the kidney weight (mg) by the rat’s body weight (g). Kidney tissues were divided, with one portion fixed in an electron microscope fixative solution and another, along with being fixed in 4% paraformaldehyde for a histopathological analysis. Ileum tissues were fixed in 4% paraformaldehyde for a histopathological analysis. Kidney tissues, ileum tissue, blood samples, and intestinal contents were promptly stored at −80 °C for a subsequent analysis.

### 2.3. Biochemical Analysis

Serum was obtained by centrifuging the blood at 1500 rpm for 15 min. Creatinine (CRE), blood urea nitrogen (BUN), and uric acid (UA) levels were measured with a Beckman AU5400 automatic biochemical analyzer (Los Angeles, CA, USA).

### 2.4. Histopathological Examination

According to a previous paper published by the research group [31], kidneys and ileums were preserved in 4% paraformaldehyde, embedded in paraffin, and sectioned into 5–6 µm slices. Hematoxylin and eosin (HE) staining and senescence-associated β-galactosidase (SA-β-gal) staining were conducted to assess kidney tissue structural damage. HE staining was used for the morphological examination of the ileum. The sections were observed and photographed with a Nikon Eclipse E100 light microscope (Nikon Corp., Tokyo, Japan). The SA-β-gal staining positive cells in the kidneys, as well as the villus height and crypt depth in the ileum, were analyzed using ImageJ software (version 1.8.0).

### 2.5. Oxidative Stress Analysis

Flow cytometry was conducted following our previously established method with minor modifications [31]. In summary, kidney tissue was processed into a single cell suspension according to the ROS assay kit instructions (Jiancheng, Nanjing, China), followed by PBS rinsing, centrifugation, and removal of the supernatant to retain the precipitate. The DCFH-DA probe was diluted and incubated for 30 min at 37 °C. The precipitate was washed with PBS, and centrifuged for collection, and its fluorescence intensity at a 488 nm laser wavelength was analyzed after resuspension in PBS. The analysis utilized a CytoFLEX flow cytometer from Beckman, CA, USA.

Commercial assay kits (Nanjing Jiancheng Corp., Nanjing, China) were used to measure CAT, SOD, GSH-PX, and MDA levels in the kidney and serum, as well as LPS levels in the serum.

### 2.6. Transmission Electron Microscopy

Kidney tissue was sectioned into 1 mm blocks; fixed in an electron microscope fixation solution, followed by 1% osmic acid solution in the dark for 2 h; dehydrated using gradient ethanol and acetone; and then embedded. Sections, 70 nm thick, were stained using 3% uranyl acetate and lead citrate. Subsequent observations were conducted with a JEM-1200EX transmission electron microscope (JEOL, Tokyo, Japan).

### 2.7. Measurement of ATP and AMP in Kidney

ATP and adenosine monophosphate (AMP) levels in kidney tissues were quantified using commercial detection kits: an ATP kit from Jiancheng, Nanjing, China, and an AMP kit from Shanghai Enzyme-Linked Biotechnology Co., Ltd. (Shanghai, China). The AMP/ATP ratio was calculated. All experimental procedures strictly followed the kit instructions.

### 2.8. Western Blot Analysis

The Western blot analysis was carried out following previous procedures with slight modification [32]. Proteins extracted from kidney tissues were separated using 10% SDS-PAGE and subsequently transferred onto PVDF membranes (Millipore, Burlington, MA, USA). The membranes were blocked with 5% skim milk powder for 2 h and incubated overnight at 4 °C with primary antibodies, including AMPK (1:1000, Boster, Pleasanton, CA, USA), P-AMPK (1:1000, Affinity, West Bridgford, UK), ULK1 (1:1000, Boster), P-ULK1 (1:1000, Affinity), Beclin-1 (1:1000, Abmart, Shanghai, China), LC3B (1:1000, Abmart), ZO-1, Claudin1 (1:1000, Cell Signaling Technology, Danvers, MA, USA), P62 (1:1000, Abmart), and β-actin (1:10000, Abmart). After washing with TBST three times, the samples were incubated with secondary antibodies (1:10000, Bioeasy, La Verne, CA, USA) for 2 h at room temperature.

### 2.9. 16S rRNA Gene Microbiome Sequencing Analysis

After 8 weeks of the experiment, the intestinal microbiome analysis of the intestinal contents of rats in the CON group, MOD group, and HF group was carried out by 16S rDNA gene sequencing technology [33]. DNA was extracted using a DNA extraction kit (Tiangen Biotech., Beijing, China), and its concentration was measured with the Qubit dsDNA HS Assay Kit and Qubit 4.0 Fluorometer (Thermo Fisher Scientific, Hillsboro, OR, USA). This included hands-on sequencing and obtaining pairs of reads with end pairs. A statistical analysis was performed on the length, distribution, and number of Clean Reads. The analysis of α-diversity and β-diversity was conducted using QIIME 2.

### 2.10. Statistical Analysis

The statistical analysis was conducted using GraphPad Prism 8.0 and SPSS 22.0. Group differences were assessed with a one-way ANOVA (when samples were normally distributed), followed by Tukey’s post hoc test for pairwise comparisons. When samples were not normally distributed, the statistical analysis was performed using the Kruskal–Wallis test (nonparametric analysis of variance) followed by Dunn’s post hoc test. Spearman’s correlation analysis was used to evaluate the relationships between kidney function indices, oxidative stress indices, and gut microbiota. Statistical significance was defined as differences with *p* < 0.05.

## 3. Results

### 3.1. Effect of FOS on Kidney Function Injury in D-Gal-Exposed Rats

**Figure 1:**
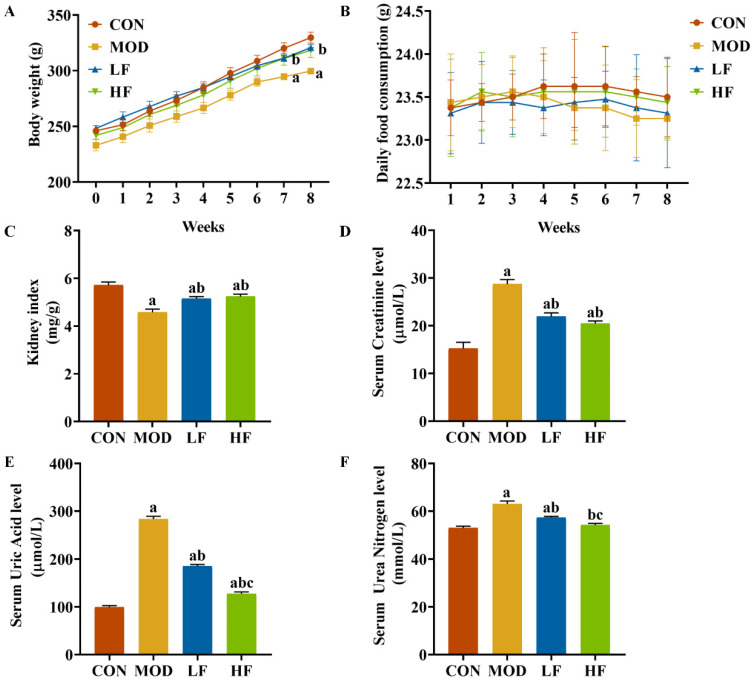
FOS attenuated D-gal-induced renal impairment. (**A**) Body weight; (**B**) daily food consumption; (**C**) kidney index; (**D**) serum creatinine; (**E**) serum uric acid; (**F**) serum urea nitrogen. All data are presented as mean ± SEM (*n* = 10). ^a^ *p* < 0.05 vs. CON group, ^b^ *p* < 0.05 vs. MOD group, ^c^ *p* < 0.05 vs. LF group.

Initially, the groups showed no significant differences in body weight. During weeks seven and eight, the MOD group exhibited lower weight compared to the CON group (Figure 1A, *p* < 0.05). FOS counteracted the weight loss in rats induced by D-gal (Figure 1A, *p* < 0.05). Throughout the experiment, all rat groups exhibited similar growth patterns, with no significant daily food consumption differences observed between them (Figure 1B, *p* = 0.314). The kidney index in the MOD group was significantly lower than that in the CON group (Figure 1C, *p* < 0.05). The FOS intervention increased the kidney index (Figure 1C, *p* < 0.05).

We assessed kidney function to evaluate kidney damage. In the MOD group, serum creatinine, serum uric acid, and serum urea nitrogen levels were significantly elevated compared to the CON group, with increases of 88.6%, 184.5%, and 19.4%, respectively (*p* < 0.05; Figure 1D–F). Intervention with FOS significantly reduced these three indicators compared to the MOD group (Figure 1D–F, *p* < 0.05). While serum creatinine levels did not significantly differ between the HF and LF groups (*p* > 0.05), the HF group exhibited notably lower serum uric acid and serum urea nitrogen levels, with reductions of 31.1% and 5.5%, respectively (Figure 1D–F, *p* < 0.05).

### 3.2. Effect of FOS on Kidney Histopathology Change in D-Gal-Exposed Rats

**Figure 2:**
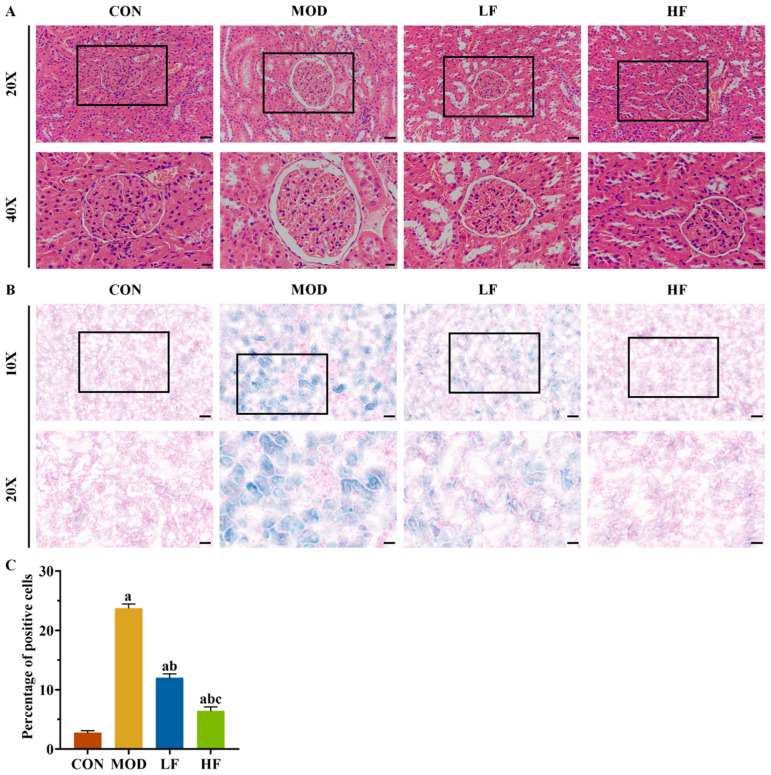
FOS alleviated D-gal-induced kidney histopathological changes and SA-β-gal staining positive cell rate. (**A**) Representative HE staining (×200, bar = 50 µm; ×400, bar = 20 µm). (**B**) Representative SA-β-gal staining (×100, bar = 100 µm; ×200, bar = 50 µm). (**C**) Percentage of SA-β-gal staining positive cells in each group. All data are presented as mean ± SEM (*n* = 3). ^a^ *p* < 0.05 vs. CON group, ^b^ *p* < 0.05 vs. MOD group, ^c^ *p* < 0.05 vs. LF group.

Aging kidneys typically undergo structural changes. Histological examination with HE staining revealed glomerular atrophy and Bowman’s capsule expansion in the MOD group (Figure 2A). Intervention with FOS was found to mitigate these phenomena (Figure 2A).

SA-β-gal staining was performed on kidney tissues to further validate the aging model (Figure 2B). Compared to the control group, the number of positive cells significantly increased in the other groups: the MOD group by 768.2%, the LF group by 339.7%, and the HF group by 134.2% (Figure 2C, *p* < 0.05). Following FOS treatment, the HF group exhibited a 46.7% reduction in positive cells compared to the LF group, as opposed to the MOD group (Figure 2C, *p* < 0.05).

### 3.3. Effect of FOS on Oxidative Stress in D-Gal-Exposed Rats

**Figure 3:**
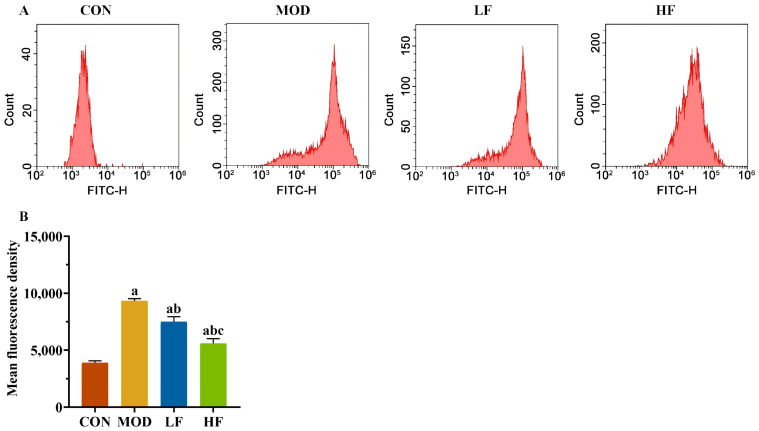
FOS declined the ROS level in D-gal-exposed rats. (**A**) The levels of ROS; (**B**) the fluorescence intensity of the probe. All data are presented as the mean ± SEM (*n* = 3). ^a^ *p* < 0.05 vs. CON group, ^b^ *p* < 0.05 vs. MOD group, ^c^ *p* < 0.05 vs. LF group.

Subsequently, we measured the levels of ROS in kidney tissues using flow cytometry (Figure 3A). The probe fluorescence intensity increased in the MOD group compared to the CON group, but decreased following FOS intervention (Figure 3B, *p* < 0.05). The fluorescence intensity of the probe was significantly lower in the HF group compared to the LF group (Figure 3B, *p* < 0.05).

**Figure 4:**
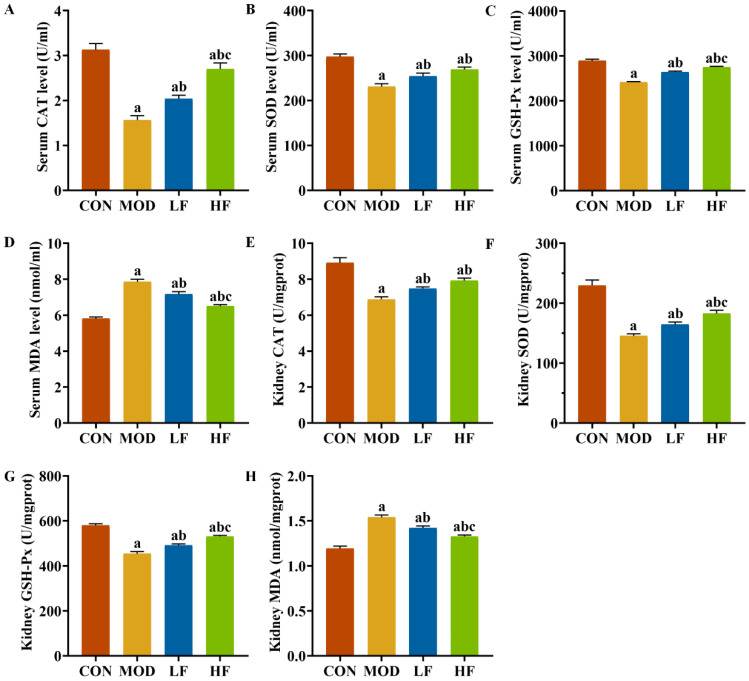
FOS attenuated oxidative stress in D-gal-exposed rats. Levels of (**A**) CAT, (**B**) SOD, (**C**) GSH-PX, and (**D**) MDA in serum; levels of (**E**) CAT, (**F**) SOD, (**G**) GSH-PX, and (**H**) MDA in kidney tissues. All data are presented as mean ± SEM (*n* = 10). ^a^ *p* < 0.05 vs. CON group, ^b^ *p* < 0.05 vs. MOD group, ^c^ *p* < 0.05 vs. LF group.

We also examined oxidative stress markers, including CAT, SOD, GSH-Px, and MDA, in both serum and kidney tissues (Figure 4A–H). The MOD group exhibited significantly lower levels of SOD, CAT, and GSH-Px compared to the CON group (*p* < 0.05). Intervention with FOS reversed the levels of these three indicators (*p* < 0.05). Additionally, the HF group showed dramatically higher levels of GSH-Px in serum (Figure 4C: *p* = 0.012) and kidney tissues (Figure 4G: *p* = 0.008) compared to the LF group (*p* < 0.05). The HF group also showed noticeably augmented levels of serum CAT (Figure 4A) and kidney tissue SOD (Figure 4F) compared to the LF group (*p* < 0.05). The MOD group exhibited higher MDA levels than the CON group (Figure 4D,H, *p* < 0.05). FOS intervention significantly decreased MDA levels (*p* < 0.05). Furthermore, the MDA levels in the HF group were lessened to a greater extent than those in the LF group (*p* < 0.05).

### 3.4. Effect of FOS on Mitochondrial Damage in D-Gal-Exposed Rats

**Figure 5:**
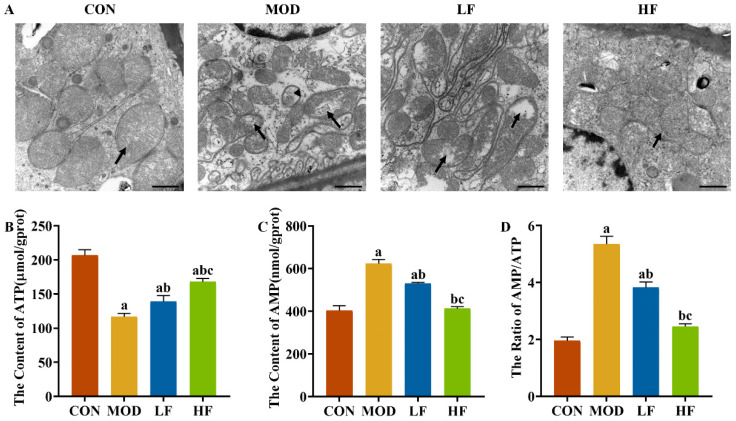
FOS improved mitochondrial damage in D-gal-exposed rats. (**A**) Representative TEM images (bar = 1 µm). Black arrow: mitochondrial cristae. Stemless arrows: mitophagosomes. Kidney ATP (**B**) and AMP (**C**) levels. AMP/ATP ratio (**D**). All data are presented as mean ± SEM (*n* = 3). ^a^ *p* < 0.05 vs. CON group, ^b^ *p* < 0.05 vs. MOD group, ^c^ *p* < 0.05 vs. LF group.

Elevated ROS levels can harm mitochondria, leading us to investigate the mitochondrial ultrastructure in the kidneys. In the CON group, the mitochondrial cristae were intact, whereas in the MOD group, the cristae were damaged, with some mitochondria showing vacuolation and autophagosomes. The intervention with FOS alleviated vacuolation and lessened the frequency of autophagosome formation (Figure 5A).

To understand the extent of mitochondrial dysfunction, we also measured ATP levels. ATP levels in the MOD group were significantly reduced by 43.6% compared to the CON group (Figure 5B, *p* < 0.05). After FOS intervention, it reversed the decrease in ATP levels caused by D-gal (Figure 5B, *p* = 0.024). Furthermore, the ATP levels in the HF group showed a 20.5% elevation compared to the levels in the LF group (Figure 5B, *p* < 0.05).

D-gal reduced ATP levels while increasing AMP levels by 54.3% and the AMP/ATP ratio by 173.7% compared to the CON group (Figure 5C,D, *p* < 0.05). FOS intervention significantly decreased AMP levels and the AMP/ATP ratio (Figure 5C,D, *p* < 0.05), with a more pronounced reduction observed in the HF group compared to the LF group (Figure 5C,D, *p* < 0.05).

### 3.5. Effect of FOS on AMPK/ULK1 Signaling Pathway in D-Gal-Exposed Rats’ Kidney Tissues

**Figure 6:**
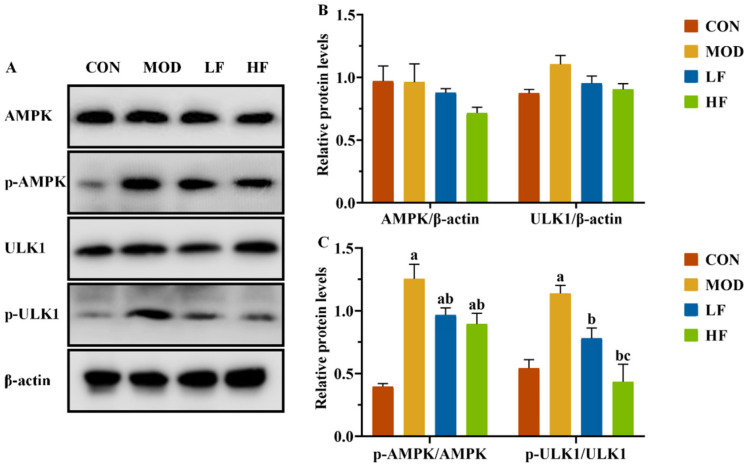
FOS downregulated p-AMPK and p-ULK1 protein levels of kidney tissue in D-gal-exposed rats. (**A**) Protein expression levels of AMPK, P-AMPK, ULK1, and P-ULK1 were measured by Western blotting; semi-quantification of (**B**) AMPK and ULK1, and (**C**) P-AMPK/AMPK and P-ULK1/ULK1, protein expression. All data are presented as mean ± SEM (*n* = 3). ^a^ *p* < 0.05 vs. CON group, ^b^ *p* < 0.05 vs. MOD group, ^c^ *p* < 0.05 vs. LF group.

It is known that changes in the AMP/ATP ratio can activate AMPK. We conducted a Western blot analysis to examine AMPK and ULK1 protein expression levels in kidney tissues (Figure 6A). The results showed no statistically substantial differences in protein expression levels among the four groups (Figure 6B, *p* > 0.05). Subsequently, we measured the expression levels of P-AMPK(Thr172) and P-ULK1(ser555). The MOD group showed significantly higher protein expression levels than the CON group (Figure 6C, P-AMPK: *p* = 0.004; P-ULK1: *p* = 0.005). Following FOS intervention, the protein expression levels of P-AMPK(Thr172) and P-ULK1(ser555), which were increased by D-gal induction, were significantly reduced (Figure 6C, *p* < 0.05).

### 3.6. Effects of FOS on Mitochondrial Autophagy-Related Proteins in D-Gal-Exposed Rats’ Kidney Tissues

**Figure 7:**
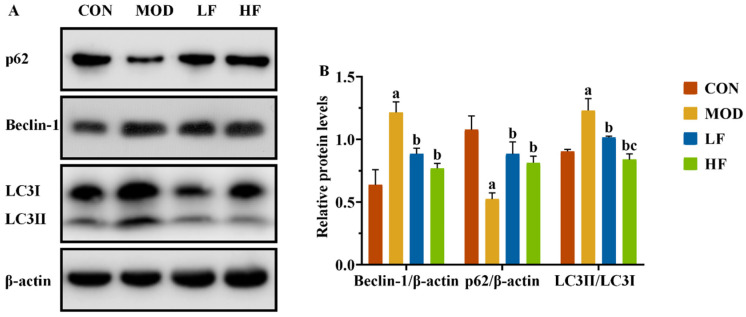
FOS inhibited excessive mitophagy by downregulating autophagy-related protein levels of kidney tissue in D-gal-exposed rats. (**A**) The protein expression levels of Beclin-1, P62, and LC3II/LC3I. (**B**) The semi-quantification of Beclin-1, P62, and LC3II/LC3I protein expression. All data are presented as the mean ± SEM (*n* = 3). ^a^ *p* < 0.05 vs. CON group, ^b^ *p* < 0.05 vs. MOD group, ^c^ *p* < 0.05 vs. LF group.

In this experiment, we observed the presence of mitophagy autophagosomes in the MOD group, while AMPK/ULK1 is also a signaling pathway that regulates mitochondrial autophagy. We also conducted a Western blot analysis to assess the expression levels of mitochondrial autophagy-related proteins Beclin-1, P62, and LC3II/LC3I (Figure 7A). The MOD group significantly increased Beclin-1 and LC3II/LC3I expression levels and decreased P62 expression compared to the CON group (Figure 7B, *p* < 0.05). Following FOS treatment, P62 expression significantly increased, while the levels of the other two proteins significantly decreased (Figure 7B, *p* < 0.05).

### 3.7. Effect of FOS on Ileum Histopathology and Tight Junction Protein Expression Levels in D-Gal-Exposed Rats

**Figure 8:**
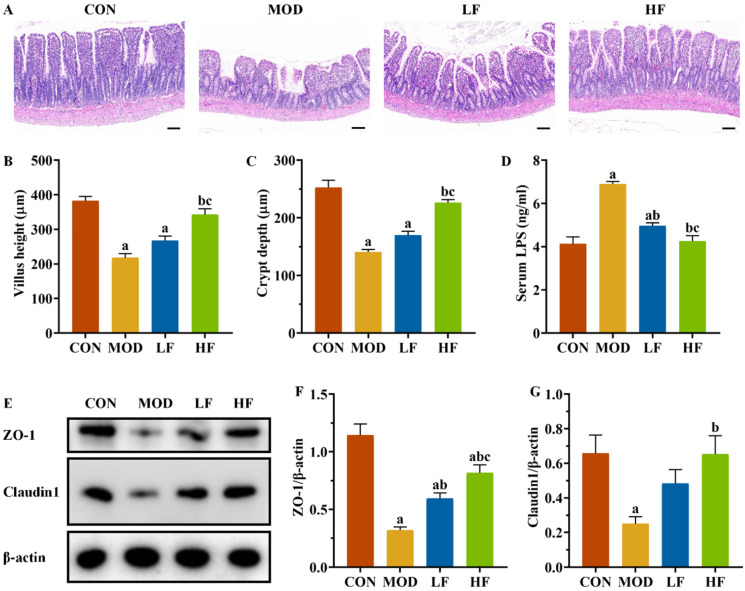
FOS improved ileal structural damage and increased tight junction protein expression levels in D-gal-exposed rats. (**A**) Pathological changes in intestine tissues by H&E staining. Scale bar = 100 µm (100×); (**B**) villus height and (**C**) crypt depth; (**D**) serum LPS level; (**E**) protein expression levels of ZO-1 and Claudin1; semi-quantification of ZO-1 (**F**) and Claudin1 (**G**) protein expression. All data are presented as mean ± SEM (*n* = 3). ^a^ *p* < 0.05 vs. CON group, ^b^ *p* < 0.05 vs. MOD group, ^c^ *p* < 0.05 vs. LF group.

The ileum in the CON group maintained its structural integrity and normal morphology. The MOD group exhibited disordered, broken, and shortened intestinal villi compared to the CON group. FOS intervention resulted in a more orderly arrangement of intestinal villi in rats, with improvements in villi height, fractures, and shedding compared to the MOD group (Figure 8A).

The MOD group exhibited significantly reduced villus height and crypt depth compared to the CON group (Figure 8B,C, *p* < 0.05). The HF group exhibited significantly greater villi height and crypt depth compared to the MOD group (*p* < 0.05). Serum LPS levels were significantly higher in the MOD group compared to the CON group (Figure 8D, *p* < 0.05), whereas both the LF and HF groups exhibited significantly lower levels (*p* < 0.05). We assessed ZO-1 and Claudin1 expression levels across groups (Figure 8E). The MOD group exhibited significantly reduced expression compared to the CON group (Figure 8F,G, *p* < 0.05), while FOS intervention led to the upregulation of these proteins (Figure 8F,G, *p* < 0.05).

### 3.8. Effects of FOS on Gut Microbiota in D-Gal-Exposed Rats

#### 3.8.1. α-Diversity Analysis3

**Figure 9:**
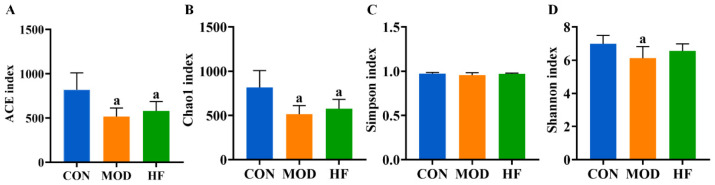
α-Diversity analysis of D-gal-exposed rats’ gut microbiota, including ACE (**A**), Chao1 (**B**), Simpson index (**C**), and Shannon index (**D**). All data are presented as mean ± SD (*n* = 6). ^a^ *p* < 0.05 vs. CON group.

The α-diversity analysis revealed that both the ACE (Figure 9A, *p* < 0.05) and Chao1 indices (Figure 9B, *p* < 0.05) were significantly reduced in the MOD and HF groups compared to the CON group. The results indicated no significant difference in intestinal flora species diversity among the groups based on the Simpson index (Figure 9C, *p* > 0.05). The Shannon index in the MOD group was significantly lower than that in the CON group (Figure 9D, *p* < 0.05).

#### 3.8.2. β-Diversity Analysis

**Figure 10:**
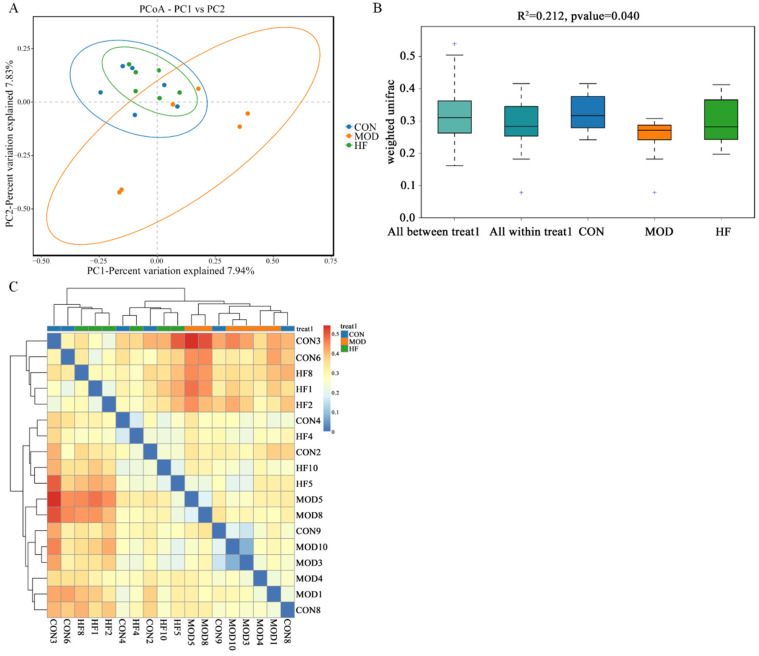
The β-diversity analysis of gut microbiota in D-gal-exposed rats. (**A**) The principal coordinate analysis between the three groups (PCoA); (**B**) ANOSIM assessed gut microbiota differences among three groups, and + shows an outlier value; (**C**) the β-diversity heatmap represents three groups’ gut microbiota variation.

According to Figure 10A, the MOD group had a notably different intestinal flora distribution than the CON group, but the HF group was similar to the CON group. This implied that D-gal greatly altered the structure and composition of the intestinal microbiota. Moreover, Figure 10B ANOSIM results indicated that the differences between groups were more pronounced than those within groups (R^2^ = 0.212, *p* = 0.040). As shown in the β-diversity heatmap (Figure 10C), the HF and CON groups exhibited greater similarity in their intestinal flora composition than the CON and MOD groups.

#### 3.8.3. Microbial Composition of Phylum and Genus Level

**Figure 11:**
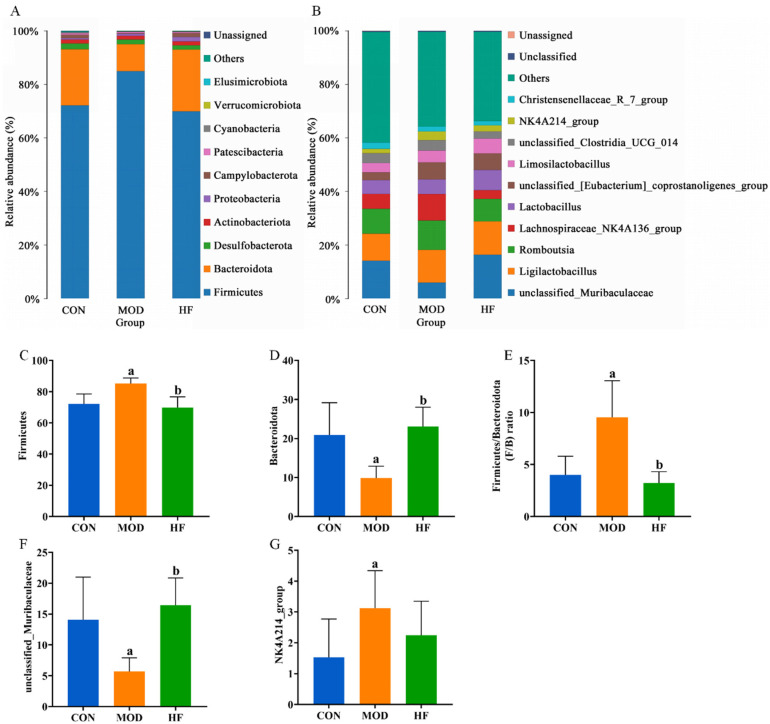
Gut microbiota community composition in each group. (**A**) Phylum; (**B**) genus; (**C**) *Firmicutes* abundance; (**D**) *Bacteroidota* abundance; (**E**) F/B ratios; (**F**) *unclassified_Muribaculaceae* abundance; (**G**) *NK4A214 group* abundance. ^a^ *p* < 0.05 vs. CON group, ^b^ *p* < 0.05 vs. MOD group.

At the phylum level, the primary constituents of the intestinal microflora are *Firmicutes*, *Bacteroidota*, *Desulfobacterota*, *Actinobacteriota*, and *Proteobacteria* (Figure 11A). Compared to the CON group, the MOD group had a significant increase in *Firmicutes* abundance, which was restored after FOS intervention (Figure 11C, *p* < 0.05). While the abundance of *Bacteroidota* was significantly lower in the MOD group compared to the CON group, it was notably higher in the HF group relative to the MOD group (Figure 11D, *p* < 0.05). The *Firmicutes*-to-*Bacteroidota* (F/B) ratios were markedly higher in the MOD group than in the CON group, but they significantly decreased after FOS was administered (Figure 11E, *p* < 0.05). Figure 11B presents the ten bacterial genera that were most abundant at the genus level. Compared to the CON group, the MOD group demonstrated a significant reduction in *unclassified_muribaculaceae* abundance, while the HF group displayed a much higher abundance than the MOD group (Figure 11F, *p* < 0.05). The MOD group exhibited a significant rise in the abundance of the *NK4A214 group* when compared to the CON group (Figure 11G, *p* < 0.05). In comparison to the MOD group, the HF group exhibited a higher abundance of *Lactobacillus* (5.8% vs. 7.5%) and a lower abundance of *Lachnospiraceae_NK4A136_group* (11% vs. 3.3%).

#### 3.8.4. Correlations Among Kidney Function-Related Indices, Oxidative Stress-Related Indices, and Gut Microbiota

**Figure 12:**
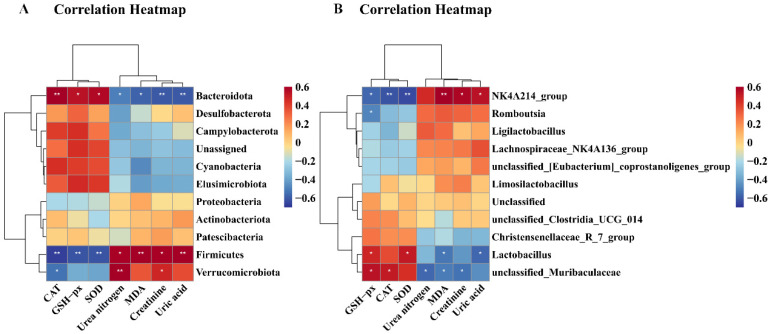
Correlation heatmap between biological index and gut microbiota. (**A**) Correlation heatmap between biological index and phylum level; (**B**) correlation heatmap between biological index and genus level. Correlations between biochemical parameters and gut microbiota are positively correlated in red, while being negatively correlated in blue. Correlations are stronger with darker colors. * *p* < 0.05 and ** *p* < 0.01 show significant correlations.

As illustrated in Figure 12, CAT exhibited a significantly positive correlation with *Bacteroidota* (*p* < 0.01) and *unclassified_Muribaculaceae* (*p* < 0.05), but correlated negatively with *Firmicutes* (*p* < 0.01), *Verrucomicrobiota* (*p* < 0.05), and *NK4A136_groups* (*p* < 0.01). GSH-Px demonstrated significantly positive correlation with *Bacteroidota* (*p* < 0.05), *unclassified_Muribaculaceae* (*p* < 0.05), and *Lactobacillus* (*p* < 0.05), while exhibiting a significantly negative correlation with *Firmicutes* (*p* < 0.01), *NK4A136_groups* (*p* < 0.05), and *Romboutsia* (*p* < 0.05). SOD correlated positively with *Bacteroidota* (*p* < 0.05), and *Lactobacillus* (*p* < 0.05), but correlated negatively with *Firmicutes* (*p* < 0.01) and *NK4A136_groups* (*p* < 0.01). MDA exhibited a significantly positive correlation with *Firmicutes* (*p* < 0.01) and *NK4A136_groups* (*p* < 0.01), but displayed a significantly negative correlation with *Bacteroidota* (*p* < 0.05), *unclassified_Muribaculaceae* (*p* < 0.05), and *Lactobacillus* (*p* < 0.05). Urea nitrogen and creatinine exhibited a significantly negative correlation with *Bacteroidota* (*p* < 0.05) and *unclassified_Muribaculaceae* (*p* < 0.05), but urea nitrogen correlated positively with *Firmicutes* (*p* < 0.05) and *Verrucomicrobiota* (*p* < 0.01), and creatinine correlated positively with *Firmicutes* (*p* < 0.05), *Verrucomicrobiota* (*p* < 0.05), and *NK4A136_groups* (*p* < 0.05).

Finally, uric acid showed a significantly negative correlation with *Bacteroidota* (*p* < 0.01) and *Lactobacillus* (*p* < 0.05), but displayed a significantly positive correlation with *Firmicutes* (*p* < 0.01) and *NK4A136_groups* (*p* < 0.05).

## 4. Discussion

Aging is a progressive, complex process influenced by a variety of internal and external factors [34]. The kidney, essential for waste excretion and blood filtration, experiences notable changes over a lifetime, such as glomerular sclerosis, tubular atrophy, arteriosclerosis, and interstitial fibrosis [35]. The D-gal-induced aging model is commonly employed in aging research for its capacity to replicate a physiological state similar to natural aging [36]. The study observed significant weight loss in rats induced by D-gal between the seventh and eighth weeks. In addition, D-gal also led to a significant decline in kidney indices. The FOS intervention restored the body weight and kidney index to their original states. A key indicator of aging is the increased activity of β-galactosidase at pH 6.0, known as SA-β-gal [37]. To further confirm the establishment of the aging model, this experiment employed the SA-β-gal staining method on kidney tissues. The number was noted that the induction by D-gal markedly elevated the quantity of positive cells, a trend which was later diminished by the application of FOS. This confirms that the experiment successfully established an aging model. This is supported by other experiments [38].

Prior studies emphasize the pivotal role of cellular aging in natural aging and kidney diseases. It is noted that the number of senescent cells escalates during physiological kidney aging and following kidney injury. Interestingly, clearing or depleting these senescent cells has been linked to improvements in age-related kidney damage and dysfunction [39]. Peng et al. discovered that FOS could normalize serum creatinine levels in rats suffering from diabetic nephropathy [40]. Abdel-Daim et al. observed that FOS effectively reduced blood urea nitrogen and creatinine levels [41]. Similarly to these experimental results, our assessment of kidney function revealed that FOS intervention reduced the elevated levels of SCr, SUA, and BUN induced by D-gal. These outcomes suggest that FOS has the potential to ameliorate kidney dysfunction induced by D-gal. Kidney aging is additionally linked with the progressive development of structural alterations, with age-related changes in kidney structure coinciding with a decline in kidney function. The HE staining results from the D-gal-induced rat model revealed glomerular atrophy and an expansion of the glomerular capsule in the kidneys. Treatment with FOS reversed the kidney glomerular atrophy and alleviated the extent of kidney glomerular capsule enlargement. These findings are supported by results from other experiments. Additionally, Sha et al. noted that D-gal induced structural abnormalities in kidney tissues in mice [42]. These findings suggest that FOS has the potential to decrease the population of senescent cells induced by D-gal and ameliorate age-related changes in kidney structure.

Aging involves multiple theories, with oxidative stress being a key pathophysiological factor in many age-related diseases [43]. In normal physiological circumstances, antioxidant enzymes can effectively neutralize hydrogen peroxide. However, the excessive administration of D-gal results in the generation of ROS. This, in turn, triggers an amplified inflammatory response and leads to mitochondrial dysfunction, culminating in a phenotype akin to natural aging in rodents [44]. ROS can inflict harm upon cellular macromolecules, encompassing lipids, proteins, and DNA, thereby precipitating both cellular and physiological aging [45]. Since ROS is a key factor in premature aging, mitigating its onset can be achieved by minimizing the accumulation of endogenous and exogenous ROS [46]. Within the cellular antioxidant system, SOD, CAT, and GSH-Px are three pivotal enzymes. Oxidative stress from ROS can cause damage that surpasses the free radical scavenging abilities of cellular antioxidant enzymes such as SOD, GSH-Px, and CAT [47]. The measurement of MDA levels can indirectly gauge the extent of cellular and organismal damage inflicted by ROS and serves as one of the indicators for assessing cellular aging [48]. We evaluated oxidative stress markers, such as ROS, MDA, SOD, CAT, and GSH-Px. The findings showed that the MOD group exhibited increased MDA and ROS levels, whereas the other three indicators were decreased. Following FOS intervention, the levels of MDA and ROS were lowered, and the decline in the remaining indicators was reversed. This is consistent with previous research findings. Our previous research demonstrated that FOS intervention mitigates alcohol-induced liver damage by reducing ROS and MDA levels, thus inhibiting oxidative stress [49]. In Ma et al.’s work, FOS was observed to boost SOD and GSH-Px activity in the kidneys, lower MDA levels, and mitigate adenine-induced kidney damage via antioxidative mechanisms [50]. These experimental results demonstrate that FOS can reduce the oxidative stress levels induced by D-gal.

Mechanistically, oxidative stress can trigger excessive ROS production, leading to mitochondrial damage [51]. Mitochondrial dysfunction leads to further ROS generation and mitochondrial impairment, creating a vicious cycle [52]. To observe mitochondrial damage, this experiment conducted transmission electron microscopy examinations on rat kidney tissues. The results showed that D-gal caused the disruption of mitochondrial cristae, along with vacuolization in some mitochondria and the presence of autophagic bodies. However, after FOS treatment, mitochondrial vacuolation was alleviated, and autophagic bodies decreased. This is consistent with previous research findings. In addition, decreased ATP production is a key indicator of mitochondrial dysfunction; our results showed that after D-galactose induction, ATP levels in rat kidneys were significantly reduced, and AMP levels and AMP/ATP ratios were significantly increased. Conversely, ATP levels are increased and AMP/ATP ratios are decreased after FOS treatment. In our earlier study, we found that pretreatment with FOS could prevent the accumulation of damaged mitochondria and excessive activation of ethanol-induced mitochondrial autophagy [53]. Wang et al. found that low-molecular-weight FOS alleviated mitochondrial dysfunction in the brains of elderly mice following traumatic brain injury [54]. Hakimizadeh et al. identified kidney pathological changes in D-gal-induced aging mice [55]. This suggests that FOS can protect against mitochondrial damage in aging kidneys.

AMPK, a serine/threonine kinase, catalyzes the phosphorylation of ULK1, which has long been thought to be a key regulator of mitophagy. Studies have shown that AMPK activation and mTOR inactivation under starvation conditions catalyze the phosphorylation of ULK1 serines at positions 317, 467, 555, 574, 637, and 777 to form multiprotein complexes, including ATG13 and FIP200, which initiate mitochondrial autophagy. The AMPK/ULK1 signaling pathway is a crucial regulator of mitochondrial autophagy. Mitochondrial autophagy is vital for maintaining kidney homeostasis, playing pivotal roles in both normal and diseased states. However, there are studies that suggest that excessive mitochondrial autophagy is involved in the occurrence of aging events [56]. We investigated the protein expression levels related to the AMPK/ULK1 signaling pathway to further understand FOS’s role in mitigating D-gal-induced oxidative stress in rat kidneys. FOS intervention notably mitigated the D-gal-induced upsurge in P-AMPK(Thr172) and P-ULK1(ser555) protein expression. These findings are consistent with those reported by Liu et al. D-gal was found to increase the expression of P-AMPK and P-ULK1 proteins in rat kidneys [57]. This indicates that FOS may safeguard against kidney aging damage by modulating the AMPK/ULK1 signaling pathway.

Microtubule-associated protein light chain 3 (LC3), the mammalian counterpart of yeast ATG8, initiates mitochondrial autophagy and includes variants LC3A, LC3B, and LC3C. Of these, LC3B is a widely recognized mitochondrial autophagy marker, while Beclin-1 is instrumental in mitochondrial autophagy initiation and autophagosome maturation [58]. The p62 proteasome serves two primary functions, facilitating the autophagic degradation of ubiquitinated proteins and mounting an antioxidative stress response, both crucial for cellular homeostasis [59]. Our research demonstrated that D-gal induction significantly increased Beclin-1 and LC3B protein levels, while markedly reducing P62 protein expression. Treatment with FOS notably reversed these changes, reducing Beclin-1 and LC3B levels and enhancing P62 expression. These findings were in line with other research. For example, Li et al. demonstrated that FOS mitigated excessive mitophagy-induced liver injury by decreasing the LC3II/LC3I ratio and Beclin-1 expression and increasing P62 levels [60]. These results suggested that FOS may offer protective effects against aging in the kidney by inhibiting excessive mitophagy.

The intestinal mucosal barrier serves as an essential defense in the body against external pathogens and toxins. Damage to the intestinal mucosal barrier increases intestinal permeability, elevating blood LPS levels and inducing oxidative stress and chronic inflammation [61,62]. Also, a previous study confirmed that LPS induces excessive mitochondrial autophagy, leading to mitochondrial dysfunction [63]. Some studies have used LPS as an inducer to induce acute kidney injury [64,65,66]. This study observed disordered, broken, and shortened ileal tissue in the MOD group, alongside increased serum LPS levels and a decreased expression of tight junction proteins. Post-FOS intervention, ileal tissue structure, serum LPS levels, and tight junction protein expression reverted to control levels.

Recent studies have increasingly highlighted the role of gut microbiota in aging. Changes in intestinal flora are intrinsically linked to kidney pathophysiology [67]. For example, intestinal microbiota product short-chain fatty acids (SCFAs) can have a renal protective role through antioxidative stress reduction, antifibrosis, and autophagy [68]. Additionally, secondary bile acids can reduce renal oxidative stress and fibrosis by activating the farnesoid X receptor (FXR) and G protein-coupled bile acid receptor 1 (TGR5) [69]. They also lower inflammation levels in the kidneys by reducing lipopolysaccharide (LPS) levels in the blood, thereby improving kidney function.

A 16S sequencing study was conducted to determine the abundance of microflora in the intestinal contents of rats in each group. It was found that *Firmicutes*’ abundance was significantly declined and *Bacteroidota*’s abundance was significantly increased after intervention with FOS compared with the MOD group. *Firmicutes* has been shown to be associated with increased intestinal permeability, intestinal mucosal damage, and increased LPS translocation [70]. The potential mechanism of FOS in reducing LPS levels may be related to the downregulation of *Firmicutes* and the increase in *Bacteroidota*. The abundance of genus *NK4A136_groups* decreased following FOS intervention compared to the MOD group. Research indicates that *NK4A136_groups* are linked to the advancement of diabetic kidney disease [71]. *Lactobacillus* is crucial for human health, particularly in maintaining intestinal and vaginal health in women. It has a variety of physiological functions, and can maintain the balance of microflora, improve digestive function, and improve immunity, in addition to affecting the progression of some diseases. Research indicates that *Lactobacillus* can decrease the urinary total protein, urinary protein/creatinine (P/C) ratio, and urine albumin excretion rate, and improve membranous nephropathy by inhibiting the aryl hydrocarbon receptor pathway [72]. In addition, studies have shown that supplementation with *Lactobacillus* can significantly reduce UA accumulation and improve renal pathological damage in mice with hyperuricemia [73]. The intervention with FOS led to an increased abundance of the genus *Lactobacillus* compared to the MOD group. FOS reduced the level of uric acid in the kidneys and improved the indicators of kidney function, which may be related to the regulation of *Lactobacillus* content. Research indicates that *Lactobacillus* can improve membranous nephropathy by inhibiting the aryl hydrocarbon receptor pathway. This could be associated with the renal protective effects of FOS [72]. These results suggest that FOS may offer protective effects against aging in the kidney by maintaining intestinal homeostasis.

## 5. Conclusions

**Figure 13:**
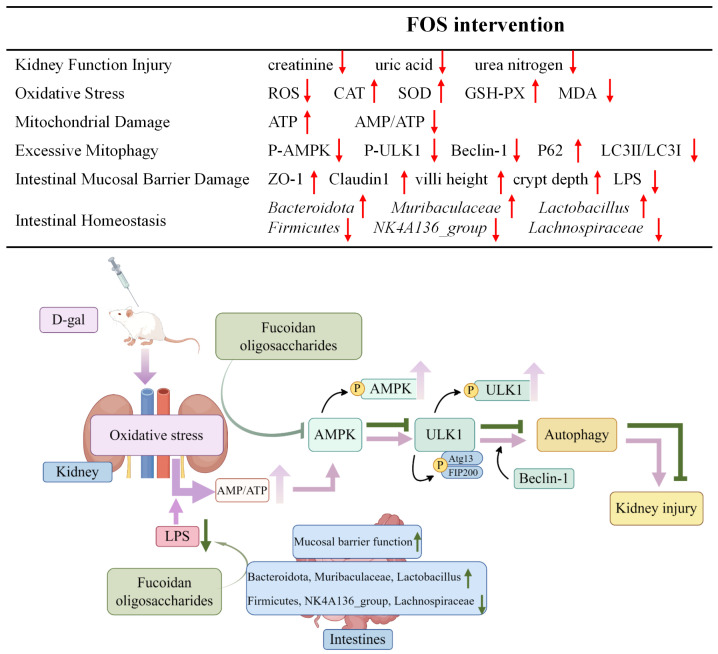
Potential alleviation mechanisms of FOS on kidney damage in D-gal-exposed rats. The upward arrow indicates increase, and the downward arrow indicates decrease.

In summary, FOS may safeguard against renal injury in D-gal-exposed rats by reducing oxidative stress, inhibiting excessive kidney mitophagy, preserving mitochondrial function, and regulating intestinal homeostasis (Figure 13). Our study offers fresh perspectives on the role of FOS in addressing D-gal-induced kidney aging-related damage; yet, it is important to acknowledge certain limitations. The use of animal models, while informative, constrains the direct applicability of our findings to human physiology. The D-galactose model also has some limitations in completely reproducing senile nephropathy, as well as the use of only one gender, which may be important. Another important limitation of this study was the short duration of the intervention. Long-term FOS supplementation may have potential effects on renal function and intestinal microbiota. Furthermore, despite the observed occurrence of mitochondrial autophagy, the precise mechanisms by which FOS influences mitophagy in the context of age-related kidney damage are not fully understood. Future research should investigate the effects of FOS in other models of kidney disease and the long-term effects of FOS supplementation, and delve into various aspects of mitophagy mechanisms, mitochondrial biogenesis, and mitochondrial dynamics, aiming to identify more potent intervention targets for the prevention of age-associated kidney damage.

## Data Availability

The original contributions presented in the study are included in the article, further inquiries can be directed to the corresponding author due to privacy reasons.
